# Intravenous medication administration by nursing staff: adherence to aseptic measures and touching environmental surfaces

**DOI:** 10.1590/0034-7167-2025-0256

**Published:** 2026-07-17

**Authors:** Patrícia de Souza Brandão Ramos, Alan Maique Ribeiro Fernandes da Costa, Ana Beatriz de Castro Vilalba, Maria Luiza Berti de Oliveira, Marcos Antonio Ferreira, Oleci Pereira Frota

**Affiliations:** I Universidade Federal de Mato Grosso do Sul. Campo Grande, Mato Grosso do Sul, Brazil

**Keywords:** Nursing, Patient Safety, Infection Control, Asepsis, Administration, Intravenous., Enfermería, Seguridad del Paciente, Control de Infecciones, Asepsia, Administración Intravenosa.

## Abstract

**Objectives:**

to analyze adherence to aseptic measures and contact with environmental surfaces by nursing professionals during intravenous medication administration.

**Methods:**

a cross-sectional study conducted in an adult Intensive Care Unit of a university hospital between February and March 2023. Twenty-seven nursing professionals were observed in a real-world care setting, and the magnitude of the associations was analyzed using the Prevalence Ratio, with a 95% Confidence Interval.

**Results:**

135 medication administration procedures were observed, and 1,083 touches on surfaces were detected (mean of eight touches per procedure). The most frequently touched surfaces were infusion pumps (29.1%), infusion sets (28.1%), and clothing (9.7%). Of the 11 indicators of compliance with aseptic measures, six were classified as non-compliant.

**Conclusions:**

low adherence to aseptic measures and a significant frequency of touching clinical surfaces before administering medications were observed, which may represent a risk to patient safety.

## RESUMEN

**Objetivos:**

analizar el cumplimiento de medidas asépticas y contacto con superficies ambientales por personal de enfermería durante administración de medicación intravenosa.

**Métodos:**

estudio transversal realizado en Unidad de Cuidados Intensivos para adultos de hospital universitario entre febrero y marzo de 2023. Hubo 27 profesionales de enfermería en un entorno de atención real, y se analizó la magnitud de las asociaciones mediante la Razón de Prevalencia, con Intervalo de Confianza del 95%.

**Resultados:**

se observaron 135 procedimientos de administración de medicamentos, y se detectaron 1.083 contactos con superficies (promedio de ocho contactos por procedimiento). Las superficies que se tocaron con mayor frecuencia fueron bombas de infusión (29,1%), equipos de infusión intravenosa (28,1%) y ropa (9,7%). De los 11 indicadores de cumplimiento de la asepsia, seis se clasificaron como incumplimientos.

**Conclusiones:**

hubo baja adherencia a medidas asépticas y una frecuencia significativa de contacto con superficies clínicas antes de administrar medicamentos, lo que puede representar un riesgo para la seguridad del paciente.

## INTRODUCTION

Healthcare-associated infections (HAIs) constitute a significant public health problem, as they increase morbidity and mortality rates, prolong hospital stays, raise healthcare costs, and promote the spread of multidrug-resistant microorganisms (MDRs)^(1,2)^. Frequently associated with the use of invasive devices, such as central vascular catheters, urinary catheters, and mechanical ventilators^(1)^, these infections are more prevalent and severe in Intensive Care Units (ICUs), given the critical clinical condition of patients admitted to these sectors^(2)^.

Critically ill patients admitted to ICUs present high rates of colonization and infection by MDR bacteria such as *Acinetobacter* sp., *Staphylococcus aureus, Staphylococcus coagulase negativa, Enterococcus* sp., *Klebsiella pneumoniae*, and *Streptococcus viridans*. Studies demonstrate that these microorganisms frequently contaminate surfaces near the bedside, making them potential reservoirs of pathogens and sources of cross-infection, especially through contact with healthcare professionals’ hands^(2-5)^. Evidence suggests that, during patient care, healthcare professionals touch environmental surfaces more frequently than patients themselves, reinforcing the role of environmental contamination in the transmission chain of HAIs^(6-8)^.

The ICU nursing staff plays a crucial role in preventing these infections, especially those associated with invasive devices such as central venous catheters^(9)^. Among the recommended safe practices to reduce the risk of infections associated with intravenous medication administration, hand hygiene before preparation and administration, use of procedure gloves, rigorous disinfection of the catheter hub with a 70% alcohol solution before handling, and continuous adoption of aseptic technique throughout the procedure stand out. These measures are supported by scientific evidence advocated by the Centers for Disease Control and Prevention and other international entities for the prevention of primary bloodstream infections associated with central vascular catheters^(10)^.

From a conceptual point of view, asepsis encompasses the set of actions aimed at preventing the introduction of microorganisms into sterile locations, such as the bloodstream or the urinary tract. Antisepsis, on the other hand, refers to measures designed to reduce or eliminate microorganisms present on the skin or inanimate surfaces through the use of substances with antimicrobial action, such as 70% alcohol^(11)^. Both are fundamental components of safe intravenous medication administration practices.

Despite the relevance of the topic, there are still few studies that systematically assess nursing professionals’ adherence to aseptic measures during intravenous medication administration, as well as the frequency of touching clinical surfaces in this context. Given this gap, this study aimed to answer: to what extent do nursing professionals adhere to aseptic measures and how frequently do they touch clinical surfaces during intravenous medication administration in an ICU?

## OBJECTIVES

To analyze adherence to aseptic measures and contact with environmental surfaces by nursing professionals during intravenous medication administration.

## METHODS

### Ethical aspects

The research was conducted in accordance with (inter)national ethical guidelines. It was approved by the *Universidade Federal de Mato Grosso do Sul* Research Ethics Committee, as per the attached opinion. All participants signed the Informed Consent Form (ICF).

### Study design, period and location

This is a cross-sectional study, guided by the Strengthening the Reporting of Observational Studies in Epidemiology (STROBE) checklist guidelines^(12)^. It was conducted in an adult ICU of a university hospital in midwestern Brazilian from February to March 2023. The hospital has 235 beds for the hospitalization of adult and pediatric patients, and is recognized as a reference center for treating infectious and parasitic diseases. The adult ICU has nine beds, two of which are reserved for respiratory isolation.

### Population, inclusion and exclusion criteria

Twenty-seven nursing professionals assigned to the aforementioned ICU who routinely administered intravenous medications were included. Professionals who were on vacation or maternity leave, on medical leave (6.1%), and those with < six months of experience in the ICU were excluded.

### Study protocol

Before beginning the observations, a researcher invited the professionals present at the site to participate in the research. Their voluntary participation was formalized ICF, and sociodemographic and occupational data were collected from the participants. Subsequently, another researcher (responsible for observing participants’ practices), who was not part of the ICU staff, spent a week familiarizing himself with the environment, which allowed him to become acquainted with the organization of routines, and professionals became accustomed to his presence^(13)^.

Data collection was carried out through direct, open, and systematic observation of nursing professionals’ practices during preparation and intravenous medication administration. The results of observations were recorded in a data collection instrument specifically designed for this purpose. A single researcher was responsible for conducting all observations in order to minimize potential disagreement between observers. Observations took place during all work shifts: morning, afternoon, and night.

The observer positioned himself preferably near the ICU counter to observe the positioning, preparation, and medication administration by professionals. However, professionals were unsure if or when they were being observed. The aseptic measures adopted before intravenous medication administration were observed. The starting point for counting touches on surfaces was the professionals’ arrival at the patients’ bedside after preparing the medications.

It is noteworthy that a single nursing professional, either a technician or a nurse, was observed a total of five times, in distinct episodes of intravenous medication administration: one episode per day, on five different days. The observations concluded when all participants had been observed. Medication administration procedures in emergencies were not the subject of this study.

Based on the pilot test (20 observations, in October 2022, at the hospital studied), carried out to benchmark quantitative variables, the following care indicators were determined: (a) percentage of adherence to hand hygiene; (b) percentage of adherence to glove use; (c) clinical surfaces most frequently touched by professionals’ hands; and (d) frequency of touching clinical surfaces by surface and overall.

The purpose of these observations was to conduct a situational diagnosis of the four care indicators in this research, without any intervention, in order to characterize the touching of surfaces and assess adherence to the observed aseptic measures.

### Analysis of results and statistics

The data were analyzed using descriptive and inferential statistics in the free statistical R version 4.2.0. The Shapiro-Wilk test was used to determine the normality of the data. Qualitative variables were compared using chi-square or Fisher’s exact tests. To estimate the magnitude of the associations, the Prevalence Ratio (PR), with a 95% Confidence Interval (95%CI), was calculated for non-parametric data. A statistically significant difference was considered when p < 0.05. Based on the theory of inductive logic, aseptic practice was considered adequate when a professional adopted the recommended measures in all five observations; otherwise, it was considered non-compliant.

## RESULTS

Of the 33 nursing professionals eligible for the study, three (9.1%) were excluded due to being on vacation, two (6.1%) due to medical leave, and one (3%) due to having less than six months of experience in the ICU. The sample consisted of 27 nursing professionals, predominantly female (66.7%), with a mean age of 40.5 ± 6.61 years. The majority were married (62.9%), held a specialist qualification (51.8%), and had a mean of 14.1 ± 4.2 years of professional nursing experience and 9 ± 5.63 years of experience working in the ICU (Table 1).

**Table 1:** Sociodemographic and occupational characterization of nursing professionals, Campo Grande, Mato Grosso do Sul, Brazil, 2023 (N=27)

Characterization	n	%
**Sex**		
Female	18	66.7
Male	9	33.3
**Age (M ± SD)**	40.48 ± 6.67	
Up to 35 years	6	22.2
Over 35 years	21	77.8
**Education**		
High school	5	18.5
Higher education	4	14.8
Specialization	14	51.9
Master’s degree	4	14.8
**Marital status**		
Married	17	63
Single	10	37
**Job title**		
Nurses	16	59.3
Nursing technicians	11	40.7
**Years of experience (years) (M ± SD)**	14.11 ± 4.24	
Up to ten years	4	14.8
Over ten years	23	85.2
**ICU experience (years) (M ± SD)**	9.04 ± 5.63	
Up to five years	8	29.6
Over five years	19	70.4
**Shift worked**		
Morning	7	25.9
Afternoon	6	22.2
Night	14	51.9

*M - mean; SD - standard deviation; ICU - Intensive Care Unit.*

**Figure 1:**
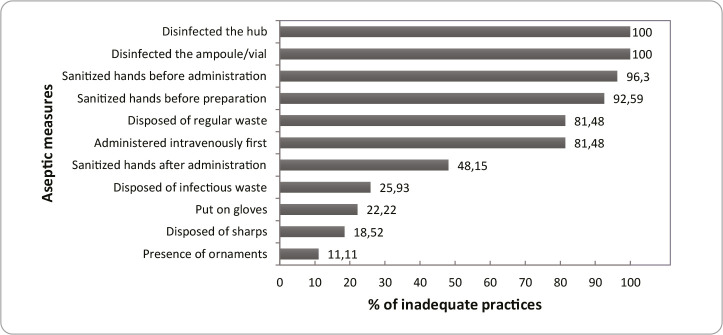
Percentage of non-compliant aseptic measures, Campo Grande, Mato Grosso do Sul, Brazil, 2023 (N=27)

In total, 135 observations were conducted during the morning, afternoon, and evening shifts. As shown in Figure 1, the aseptic measures with the highest percentages of compliance were presence of ornaments (88.8%) and proper disposal of sharps (81.5%). It is noteworthy that no professional adopted appropriate conduct in the five observation episodes regarding the following aseptic measures: ampoule or vial disinfection and hub disinfection. More than 80% of participants practiced six aseptic measures non-compliantly. As for ampoule or vial disinfection and hub disinfection before intravenous administration, all professionals performed these actions non-compliantly.

**Figure 2:**
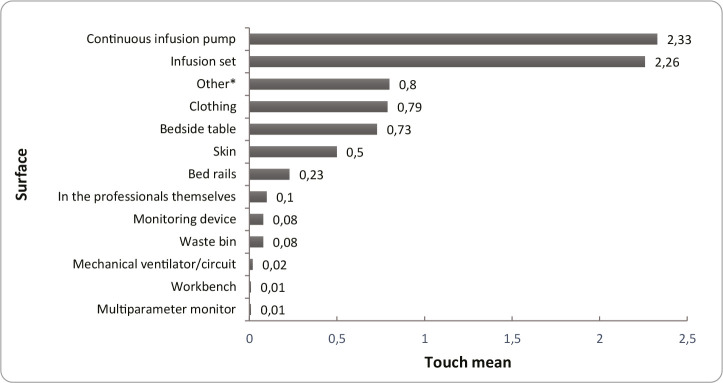
Mean number of touches per surface, Campo Grande, Mato Grosso do Sul, Brazil, 2023 (N=27)

A total of 135 observations were made regarding contact with surfaces, of which nine did not record contact with any surface or the patients’ skin. In total, 1,083 episodes of touching by healthcare professionals’ hands were recorded, with the most frequently touched surfaces being infusion pump (29.1%), infusion sets (28.1%), clothing (9.7%), and bedside table (9.1%). Other contact locations accounted for 108 touches (10%), and included alcohol bottles, water bottles, chlorhexidine bottles, glucometers, blood glucose strips, lancets, tracheostomy tubes, nasoenteral tubes, feeding bottles, pens, cell phones, light switches, and clipboards. Two surfaces had the highest overall average number of touches: continuous infusion pump (2.33) and infusion sets (2.26); together they account for 57.2% of the total frequency of observed touches. The surfaces with the lowest mean number of touches were the multiparameter monitor (0.01), countertop (0.01), and mechanical ventilator (0.02). The mean number of touches on clinical surfaces per medication administration episode was 8.02 times (Figure 2).

*Others: alcohol bottle, water bottle, chlorhexidine bottle, glucometer, blood glucose test strip, lancet, tracheostomy, nasoenteral tube, feeding bottle, pen, cell phone, light switch, clipboard, and gauze pack.

The 11 indicators of compliance with aseptic measures presented in Figure 1 were analyzed in relation to participants’ sociodemographic and occupational variables. Two statistically significant associations were identified: the prevalence of adequate glove use was 4.86 times higher among female professionals (PR: 4.86; 95%CI: 0.80-29.42; p=0.008), and professionals working the night shift showed a higher level of non-compliance with the aseptic measures analyzed (p=0.028) when compared to their counterparts (Table 2).

**Table 2:** Aseptic measures according to sociodemographic and occupational data, Campo Grande, Mato Grosso do Sul, Brazil, 2023 (N=27)

Characterization	Put on gloves		*p* value	Prevalence Ratio [95%CI]
	According to	Non-compliant		
**Sex**			**0.008**†	**4.86 [0.80 ; 29.42]**
Female	94.4(17)	5.6(1)		
Male	44.4(4)	55.6(5)		
**Age**			1.000†	1.43 [0.20 ; 10.00]
≤ 35 years	83.3(5)	16.7(1)		
> 35 years	76.2(16)	23.8(5)		
**Marital status**			0.638†	1.33 [0.57 ; 3.14]
Married	82.4(14)	17.7(3)		
Single	70(7)	30(3)		
**Role**			0.662†	1.24 [0.52 ; 2.95]
Nurses	81.3(13)	18.8(3)		
Nursing technicians	72.7(8)	27.3(3)		
**Years of experience**			1.000†	0.86 [0.11 ; 6.82]
≤ ten years	75(3)	25.(1)		
> ten years	78.3(18)	21.7(5)		
**ICU stay**			1.000†	0.86 [0.23 ; 3.20]
≤ five years	75(6)	25(2)		
> five years	79(15)	21.1(4)		
**Shift that operates**			0.028^*^	**---**
Morning	100(7)	---		
Evening	57.1(8)	42.9(6)		
Afternoon	100(6)	---		

* *Chi-square test, †Fisher’s exact test; 95%CI - 95% Confidence Interval; ICU - Intensive Care Unit.*

## DISCUSSION

The results of this research provide valuable information on adherence to aseptic practices and the frequency of surface contact in hospital settings. Analysis of the observations reveals some important findings that deserve discussion in light of existing literature and best infection control practices.

Traditionally, efforts to prevent hospital-acquired infections have focused primarily on hand hygiene. From the 1970s onwards, clinical surfaces began to be seen as potential reservoirs of pathogenic microorganisms and, therefore, could facilitate the occurrence of cross-infections^(14)^. Although patient safety studies focusing on medication administration are widespread, this is the first study to assess the frequency of touching clinical surfaces by nursing professionals in the context of intravenous medication administration in critically ill patients^(15-17)^.

The results obtained in this study reveal a worrying gap in adherence to aseptic measures among the professionals observed. Hands are the main vehicles for the spread of pathogens in the healthcare sector, contributing to HAI development. The presence of ornaments showed a high compliance rate (88.8%), which suggests good adherence by professionals to the institutional policy of zero ornaments and awareness of the risk of pathogen transmission associated with the use of accessories.

Conversely, in over 90% of administrations, professionals did not sanitize their hands before preparing and administering medications. In this regard, the World Health Organization emphasizes the importance of performing hand disinfection properly and safely, which should always follow the five moments when hand hygiene is essential^(18)^. Therefore, negligence in hand hygiene in other practices also raises concerns, especially in situations involving invasive care. The Brazilian National Health Regulatory Agency recommends the use of alcohol-based hand sanitizers before putting on gloves for non-surgical procedures, as well as before performing care procedures and handling invasive devices^(19,20)^.

Microbiological safety is compromised when nursing staff fail to perform essential aseptic measures during medication administration, such as hand hygiene and disinfection of vials and ampoules^(21)^. In this context, the nurse has a crucial role in implementing continuous training strategies for their staff. It is essential to maintain a vigilant and proactive stance, mobilizing collective efforts to reduce risks and prevent failures in healthcare.

It was also observed that no professional adopted appropriate conduct in the five observed episodes regarding ampoule or vial aseptic disinfection measures and catheter hub disinfection. This finding is concerning, considering that inadequate handling of these components can favor the introduction of microorganisms into the bloodstream, contributing to the development of primary bloodstream infections and sepsis. Evidence suggests that failure to adequately disinfect the catheter hub is among the main risk factors for infections associated with central venous catheters, being considered a critical step in the prevention of this type of infection^(10)^.

Inadequate disinfection of these surfaces can be a critical point in controlling infections associated with intravenous care, such as catheter-related infections. The literature indicates that proper disinfection of vials and materials used in invasive procedures is essential to reduce the risk of infection, and the absence of proper practices suggests a significant gap in infection control practices in this setting^(21)^.

In turn, more than 80% of participants performed six aseptic measures incorrectly, raising concerns about healthcare professionals’ adherence to infection control recommendations. More frequent training and audits may be necessary to improve adherence to these protocols.

It is generally agreed in the literature that clinical surfaces in ICUs are potentially contaminated by various pathogens, such as vancomycin-resistant *Enterococcus*, methicillin-resistant *S. aureus, Clostridium difficile*, and *Acinetobacter* sp., microorganisms capable of persisting in the environment even after terminal cleaning of the beds^(3)^. A prospective study using microbiological methods revealed the presence of microorganisms in 81.1% of samples collected after terminal cleaning in ICU beds, with 38.8% being Gram-negative bacilli^(2)^. Therefore, it can be stated that surfaces are potentially colonized by pathogens and inferred that touching clinical surfaces by healthcare professionals can increase the risk of cross-infection during hospitalization^(22)^.

In this study, 1,083 instances of healthcare professionals touching environmental surfaces with their hands were recorded, most frequently involving critical devices such as infusion pumps (29.1%) and infusion sets (28.1%), which together accounted for more than half of the observed contacts. However, more important than the absolute number of touches is that many of these contacts occurred without the adoption of recommended aseptic precautions, which can significantly increase the risk of cross-contamination during intravenous drug administration.

The average number of touches on clinical surfaces per administration episode was 8 times, a relatively high value that points to the medication environment as a critical point for pathogen transmission. This setting reinforces the need for rigorous control policies, with an emphasis on the sanitization of handled surfaces and on raising awareness among professionals about proper practices.

The high frequency of touching surfaces near the patient can be explained by several factors, including work overload, which can compromise adherence to recommended protocols, hindering the correct execution of infection prevention measures^(23)^. Furthermore, insufficient knowledge about aseptic practices also contributes to this reality. A study conducted in an ICU with medical and nursing staffs revealed limited knowledge regarding the gold standard measures for preventing central venous catheter-related bloodstream infections^(24)^.

The discrepancies in aseptic measures observed are greater than those found in a study conducted in Morocco^(25)^, which revealed non-compliance with aseptic standards. In this study, 14.3% of professionals did not sanitize their hands before and after procedures; 7% did not use gloves during patient care; and 5.6% did not change gloves between treatments. These practices can favor the occurrence of hospital-acquired infections, worsen the clinical condition of patients, increase hospitalization costs, and raise the risk of death during the hospital stay^(2,15)^.

Despite the high level of professional experience found in this study, low adherence to recommended practices for infection prevention was observed. It is necessary to invest in strategies that raise awareness among the staff regarding the importance of adhering to aseptic measures and the role of environmental surfaces in the transmission chain of microorganisms.

### Study limitations

This study was limited by its sample size and the fact that it was conducted in a single ICU of a specific university hospital, which restricts the generalizability of the results to other units or healthcare settings. Despite mitigation measures, the “Hawthorne Effect”-the effect of observation on participants’ behavior, characterized as reactivity bias-may have occurred. Thus, it is inferred that participants’ practice may be of lower quality than observed in the results. To minimize this effect, professionals were observed during five procedures, on five different days, by an independent observer not belonging to the service.

Therefore, future research should explore the impact of multimodal interventions, including logistical, educational, and organizational restructuring, as well as quality improvement programs that address the problem of clinical surfaces as potential reservoirs of microorganisms. Furthermore, it is important to implement compliance indicators in intravenous medication administration. This research should also consider regional characteristics and needs, with larger samples, in diverse locations, and with quasi-experimental designs.

### Contributions to nursing and health

The results of this study support the implementation of quality improvement programs. It is recommended to expand continuing education policies, review institutional protocols, provide ongoing staff training, and monitor and provide feedback on the results^(26)^. Furthermore, the strategic role of the staff leader nurse in promoting adherence to evidence-based practices and in consolidating a culture of safety in the care provided by the nursing staff is highlighted.

## CONCLUSIONS

This study revealed that several essential safety barriers for HAI prevention showed non-compliance during intravenous medication administration by nursing professionals, which can compromise patient safety and result in serious complications. The implementation of a care management program and periodic assessments of medication administration practices are suggested. In this way, it is possible to contribute to increased adherence to aseptic measures and reduce the frequency of unnecessary touching of potentially contaminated surfaces during medication administration and other nursing procedures.

## Data Availability

The research data are available only upon request.
