# Supplementary material for: Intravenous medication administration by nursing staff: adherence to aseptic measures and touching environmental surfaces
Source: Rev Bras Enferm. 2026 Jul 17;79:e20250256. doi: 10.1590/0034-7167-2025-0256 (PMC13379196; doi:10.1590/0034-7167-2025-0256)
Supplement: Translation

# Translation: Administração intravenosa de medicamentos pela enfermagem: adesão às medidas assépticas e toque em superfícies ambientais

**DOI:** 10.1590/0034-7167-2025-0256pt

**Authors:** Patrícia de Souza Brandão Ramos, Alan Maique Ribeiro Fernandes da Costa, Ana Beatriz de Castro Vilalba, Maria Luiza Berti de Oliveira, Marcos Antonio Ferreira, Oleci Pereira Frota

**Affiliations:** I Universidade Federal de Mato Grosso do Sul. Campo Grande, Mato Grosso do Sul, Brasil

**Keywords:** Enfermagem, Segurança do Paciente, Controle de Infecções, Assepsia, Administração Intravenosa.

## Abstract

**Objetivos:**

analisar a adesão às medidas assépticas e o toque nas superfícies ambientais pelos profissionais de enfermagem na administração endovenosa de medicamentos.

**Métodos:**

estudo transversal realizado em Unidade de Terapia Intensiva adulto de um hospital universitário entre fevereiro e março de 2023. Vinte e sete profissionais de enfermagem foram observados em situação real de assistência, cuja magnitude das associações foi analisada pela Razão de Prevalência, com Intervalo de Confiança de 95%.

**Resultados:**

foram observados 135 procedimentos de administração de medicamentos e detectados 1.083 toques em superfícies (média de oito toques por procedimento). As mais tocadas foram bomba de infusão (29,1%), equipos (28,1%) e roupas (9,7%). Dos 11 indicadores de conformidade de medidas assépticas, seis foram classificados como não conforme.

**Conclusões:**

verificaram-se baixa adesão às medidas assépticas e frequência expressiva de toques em superfícies clínicas antes da administração de medicamentos, o que pode representar risco à segurança do paciente.

## INTRODUÇÃO

As infecções relacionadas à assistência à saúde (IRAS) constituem importante problema de saúde pública, uma vez elevam as taxas de morbimortalidade, prolongam o tempo de internação, aumentam os custos assistenciais e favorecem a disseminação de microrganismos multirresistentes (MMR)^(1,2)^. Frequentemente associadas ao uso de dispositivos invasivos, como cateteres vasculares centrais, cateteres urinários e ventiladores mecânicos^(1)^, essas infecções apresentam maior incidência e gravidade nas Unidades de Terapia Intensiva (UTIs), devido à criticidade clínica dos pacientes internados nesses setores^(2)^.

Pacientes críticos internados em UTIs apresentam elevadas taxas de colonização e infecção por MMR, como *Acinetobacter* sp., *Staphylococcus aureus, Staphylococcus coagulase negativa, Enterococcus* sp., *Klebsiella pneumoniae* e *Streptococcus viridans*. Estudos demonstram que tais microrganismos frequentemente contaminam superfícies próximas ao leito, tornando-as potenciais reservatórios de patógenos e fontes de infecção cruzada, especialmente por meio do contato com as mãos dos profissionais de saúde^(2-5)^. Evidências apontam que, durante a assistência, os profissionais tocam mais frequentemente superfícies ambientais do que o próprio paciente, o que reforça o papel da contaminação ambiental na cadeia de transmissão das IRAS^(6-8)^.

A equipe de enfermagem da UTI desempenha papel fundamental na prevenção dessas infecções, especialmente daquelas associadas a dispositivos invasivos, como os cateteres venosos centrais^(9)^. Entre as práticas seguras recomendadas para reduzir o risco de infecções associadas à administração intravenosa de medicamentos, destacam-se a higienização das mãos antes do preparo e da administração, o uso de luvas de procedimento, a desinfecção rigorosa do *hub* do cateter com solução alcoólica a 70% antes da manipulação, e a adoção contínua de técnica asséptica durante todo o procedimento. Essas medidas são sustentadas por evidências científicas preconizadas pelo *Centers for Disease Control and Prevention* e por outras entidades internacionais para prevenção de infecções primárias de corrente sanguínea associadas a cateteres vasculares centrais^(10)^.

Do ponto de vista conceitual, a assepsia compreende o conjunto de ações voltadas à prevenção da introdução de microrganismos em locais estéreis, como a corrente sanguínea ou o trato urinário. Já a antissepsia se refere às medidas destinadas à redução ou eliminação de microrganismos presentes na pele ou em superfícies inanimadas mediante o uso de substâncias com ação antimicrobiana, como o álcool 70%^(11)^. Ambas são componentes fundamentais das práticas seguras de administração de medicamentos por via endovenosa.

Apesar da relevância do tema, ainda são escassos os estudos que avaliem, de forma sistemática, a adesão dos profissionais de enfermagem às medidas assépticas durante a administração intravenosa de medicamentos, bem como a frequência de toques em superfícies clínicas nesse contexto. Diante dessa lacuna, este estudo propõe-se a responder: em que medida os profissionais de enfermagem aderem às medidas assépticas e com que frequência realizam toques em superfícies clínicas durante a administração intravenosa de medicamentos em uma UTI?

## OBJETIVOS

Analisar a adesão às medidas assépticas e o toque nas superfícies ambientais pelos profissionais de enfermagem na administração endovenosa de medicamentos

## MÉTODOS

### Aspectos éticos

A pesquisa foi realizada segundo as diretrizes éticas (inter)nacionais. Foi aprovada pelo Comitê de Ética em Pesquisa da Universidade Federal de Mato Grosso do Sul, conforme parecer anexado. Todos os participantes assinaram o Termo de Consentimento Livre e Esclarecido (TCLE).

### Desenho, período e local do estudo

Estudo transversal, norteado pelas diretrizes do *checklist Strengthening the Reporting of Observational Studies in Epidemiology* ^(12)^, realizado em UTI adulto de um hospital universitário do centro-oeste brasileiro de fevereiro a março de 2023. O hospital conta com 235 leitos destinados à internação de pacientes adultos e pediátricos, sendo reconhecido como referência no tratamento de doenças infecto-parasitárias. A UTI adulto dispõe de nove leitos, dos quais dois são reservados para isolamento respiratório.

### População, critérios de inclusão e exclusão

Foram incluídos 27 profissionais de enfermagem lotados na referida UTI que realizavam administração endovenosa de medicamentos na sua rotina. Foram excluídos profissionais que estavam de férias ou licença maternidade, atestado médico (6,1%) e aqueles com tempo de atuação em UTI < seis meses.

### Protocolo do estudo

Antes de iniciar as observações, um pesquisador convidou os profissionais *in loco* para participar da pesquisa, cuja participação voluntária foi formalizada por TCLE, sendo coletados os dados sociodemográficos e ocupacionais dos participantes. Na sequência, outro pesquisador (responsável pela observação das práticas dos participantes) não integrante da equipe da UTI passou um período de uma semana de ambientação, o que lhe permitiu se familiarizar com a organização das rotinas, e os profissionais se habituaram à sua presença^(13)^.

A coleta de dados foi realizada mediante observação direta, aberta e sistemática das práticas dos profissionais de enfermagem durante o preparo e administração endovenosa de medicamentos. O resultado das observações foi registrado em instrumento de coleta de dados especificamente confeccionado para esta finalidade. Um único pesquisador foi responsável por realizar todas as observações, com vistas a minimizar a eventual discordância entre observadores. As observações ocorreram em todos os turnos de trabalho: manhã, tarde e noite.

O observador se posicionou preferencialmente próximo à bancada da referida UTI para realizar as observações desde o posicionamento, preparo e administração das medicações pelos profissionais. Para tanto, os profissionais não sabiam ao certo se e quando eram observados. Foram observadas as medidas assépticas adotadas antes da administração endovenosa de medicamentos. O ponto de partida para início da contagem do toque nas superfícies foi a chegada do profissional ao leito do paciente após o preparo dos medicamentos.

Destaca-se que um único profissional de enfermagem, sendo técnico ou enfermeiro, foi observado, em um total de cinco vezes, em episódios distintos de administração endovenosa de medicamentos: um episódio por dia, em cinco dias distintos. As observações finalizaram quando todos os participantes foram observados. Procedimentos de administração de medicamentos em emergências não foram objeto deste estudo.

A partir do teste piloto (20 observações, em outubro de 2022, no hospital pesquisado), realizado para balizar as variáveis quantitativas, foram determinados os seguintes indicadores assistenciais: (a) percentual de adesão à higienização das mãos; (b) percentual de adesão ao uso de luvas; (c) superfícies clínicas mais frequentemente tocadas pelas mãos dos profissionais; e (d) frequência de toque nas superfícies clínicas por superfície e geral.

A finalidade dessas observações foi realizar um diagnóstico situacional dos quatro indicadores assistenciais desta pesquisa, sem qualquer intervenção, a fim de caracterizar o toque nas superfícies e avaliar a adesão às medidas assépticas observadas.

### Análise dos resultados e estatística

Os dados foram analisados por estatística descritiva e inferencial no *software* estatístico livre R, versão 4.2.0. O teste de Shapiro-Wilk foi empregado para determinar a normalidade dos dados. As variáveis qualitativas foram comparadas por meio dos testes qui-quadrado ou exato de Fisher. Para estimar a magnitude das associações, a Razão de Prevalência (RP), com Intervalo de Confiança de 95% (IC95%), foi calculada nos dados não paramétricos. Diferença estatisticamente significativa foi considerada quando p <0,05. Sustentado na teoria da lógica indutiva, a prática asséptica foi considerada adequada quando o profissional adotou as medidas recomendadas em todas as cinco observações; caso contrário, foi considerada não conforme.

## RESULTADOS

Dos 33 profissionais de enfermagem elegíveis para a pesquisa, foram excluídos três (9,1%) por estarem em período de férias, dois (6,1%), por afastamento médico, e um (3%), por tempo de atuação em UTI inferior a seis meses. A amostra foi composta por 27 profissionais de enfermagem, predominantemente do sexo feminino (66,7%), com média de idade de 40,5 ± 6,61 anos. A maioria era casada (62,9%), possuía titulação de especialista (51,8%) e apresentava média de 14,1 ± 4,2 anos de experiência profissional em enfermagem e 9 ± 5,63 anos de atuação em UTI (Tabela 1).

**Tabela 1:** Caracterização sociodemográfica e ocupacional dos profissionais de enfermagem, Campo Grande, Mato Grosso do Sul, Brasil, 2023 (N=27)

Caracterização	n	%
**Sexo**		
Feminino	18	66,7
Masculino	9	33,3
**Idade** (M ± DP)	40,48 ± 6,67	
Até 35 anos	6	22,2
Acima de 35 anos	21	77,8
**Formação**		
Ensino médio	5	18,5
Ensino superior	4	14,8
Especialização	14	51,9
Mestrado	4	14,8
**Estado civil**		
Casado	17	63
Solteiro	10	37
**Função**		
Enfermeiros	16	59,3
Técnico de enfermagem	11	40,7
**Tempo de experiência (anos)** (M ± DP)	14,11 ± 4,24	
Até dez anos	4	14,8
Acima de dez anos	23	85,2
**Tempo de UTI (anos)** (M ± DP)	9,04 ± 5,63	
Até cinco anos	8	29,6
Acima de cinco anos	19	70,4
**Turno que atua**		
Matutino	7	25,9
Vespertino	6	22,2
Noturno	14	51,9

*M - média; DP - desvio padrão; UTI - Unidade de Terapia Intensiva.*

Ao todo, foram realizadas 135 observações nos turnos matutino, vespertino e noturno. Conforme a Figura 1, as medidas assépticas que obtiveram maiores porcentagens de adequação foram presença de adornos (88,8%) e descarte de perfuro cortante em local correto (81,5%). Destaca-se que nenhum profissional adotou conduta adequada nos cinco episódios de observação nas seguintes medidas assépticas: desinfecção da ampola ou frasco-ampola e desinfecção do *hub*. Mais de 80% dos participantes praticam seis medidas assépticas de maneira não conforme. Em relação às medidas de desinfecção da ampola ou frasco-ampola e de desinfecção do *hub* antes da administração endovenosa, todos os profissionais realizam essas ações de forma não conforme.

**Figura 1:** Percentual de medidas assépticas em não conformidade. Campo Grande, Mato Grosso do Sul, Brasil, 2023 (n=27)

Foram realizadas 135 observações relativas ao toque nas superfícies, das quais nove não registraram contato com nenhuma superfície ou pele do paciente. Ao todo, contabilizaram-se 1.083 episódios de toque pelas mãos dos profissionais, cujas superfícies mais frequentemente tocadas foram bomba de infusão (29,1%), equipos (28,1%), roupas (9,7%) e mesa de cabeceira (9,1%). Outros locais de toque corresponderam a 108 toques (10%), e abrangeram frasco de álcool, garrafa de água, frasco de clorexidina, glicosímetro, fita de glicemia, lanceta, traqueostomia, sonda nasoenteral, frasco de dieta, caneta, celular, interruptor e prancheta. Duas superfícies possuem as maiores médias gerais de toque: bomba de infusão contínua (2,33) e equipos (2,26); juntas representam 57,2% da frequência total de toques observados. As superfícies que possuem as menores médias de toques foram monitor multiparamétrico (0,01), bancada (0,01) e ventilador mecânico (0,02). A média de toques em superfícies clínicas por episódio de administração de medicação foi de 8,02 vezes (Figura 2).

**Figura 2:** Média de toque por superfície, Campo Grande, Mato Grosso do Sul, Brasil, 2023 (N=27)

*Outros: frasco de álcool, garrafa de água, frasco de clorexidina, glicosímetro, fita de glicemia, lanceta, traqueostomia, sonda nasoenteral, frasco de dieta, caneta, celular, interruptor, prancheta e pacote de gaze.

Os 11 indicadores de conformidade com medidas assépticas apresentados na Figura 1 foram analisados em relação às variáveis sociodemográficas e ocupacionais dos participantes. Duas associações estatisticamente significantes foram identificadas: a prevalência de uso adequado de luvas foi 4,86 vezes maior entre profissionais do sexo feminino (RP: 4,86; IC95%: 0,80-29,42; p=0,008) e profissionais do turno noturno apresentaram maior nível de não conformidade nas medidas assépticas analisadas (p=0,028), quando comparados aos seus homólogos (Tabela 2).

**Tabela 2:** Medidas assépticas segundo dados sociodemográficos e ocupacionais, Campo Grande, Mato Grosso do Sul, Brasil, 2023 (N=27)

Caracterização	Calçou luvas		Valor de *p*	Razão de Prevalência [IC95%]
	Conforme	Não conforme		
**Sexo**			**0,008**†	**4,86 [0,80 ; 29,42]**
Feminino	94,4(17)	5,6(1)		
Masculino	44,4(4)	55,6(5)		
**Idade**			1,000†	1,43 [0,20 ; 10,00]
≤ 35 anos	83,3(5)	16,7(1)		
> 35 anos	76,2(16)	23,8(5)		
**Estado civil**			0,638†	1,33 [0,57 ; 3,14]
Casado	82,4(14)	17,7(3)		
Solteiro	70(7)	30(3)		
**Função**			0,662†	1,24 [0,52 ; 2,95]
Enfermeiros	81,3(13)	18,8(3)		
Técnico de enfermagem	72,7(8)	27,3(3)		
**Tempo de experiência**			1,000†	0,86 [0,11 ; 6,82]
≤ dez anos	75(3)	25,(1)		
> dez anos	78,3(18)	21,7(5)		
**Tempo de UTI**			1,000†	0,86 [0,23 ; 3,20]
≤ cinco anos	75(6)	25(2)		
> cinco anos	79(15)	21,1(4)		
**Turno que atua**			0,028^*^	**---**
Matutino	100(7)	---		
Noturno	57,1(8)	42,9(6)		
Vespertino	100(6)	---		

* *Teste qui-quadrado; †Teste exato de Fisher; IC95% - Intervalo de Confiança de 95%; UTI - Unidade de Terapia Intensiva.*

## DISCUSSÃO

Os resultados desta pesquisa fornecem informações valiosas sobre o cumprimento das práticas assépticas e da frequência de toques em superfícies no ambiente hospitalar. A análise das observações realizadas revela algumas descobertas importantes que merecem ser discutidas à luz da literatura existente e das melhores práticas de controle de infecções.

Tradicionalmente, os esforços de prevenção à infecção hospitalar se concentraram majoritariamente na higienização das mãos. A partir dos anos 70, as superfícies clínicas passaram a figurar como potenciais reservatórios de microrganismos patogênicos, podendo, portanto, propiciar a ocorrência de infecções cruzadas^(14)^. Embora estudos de segurança do paciente com foco na administração de medicamentos sejam amplamente difundidos, este é o primeiro estudo a avaliar a frequência de toque em superfícies clínicas por profissionais de enfermagem no contexto da administração de medicamentos via endovenosa em pacientes críticos^(15-17)^.

Os resultados obtidos neste estudo revelam uma lacuna preocupante nas práticas de adesão às medidas assépticas entre os profissionais observados. As mãos são os principais veículos de propagação de patógenos no setor assistencial, contribuindo para o desenvolvimento de IRAS. A presença de adornos apresentou alta taxa de conformidade (88,8%), o que sugere boa adesão dos profissionais à política institucional de adorno zero e à sensibilização sobre o risco de transmissão de patógenos associados ao uso de acessórios.

Em contrapartida, em mais de 90% das administrações, o profissional não higienizou as mãos antes do preparo e da administração de medicamentos. Nesse sentido, a Organização Mundial da Saúde enfatiza a importância de realizar a desinfecção das mãos de forma adequada e segura, que deve sempre seguir os cinco momentos em que a higiene das mãos é imprescindível^(18)^. Portanto, a negligência na higienização das mãos em outras práticas também levanta preocupações, especialmente em situações que envolvem cuidados invasivos. A Agência Nacional de Vigilância Sanitária recomenda o uso de soluções alcoólicas antes de calçar luvas para procedimentos não cirúrgicos, assim como antes de realizar procedimentos assistenciais e manipular dispositivos invasivos^(19,20)^.

A segurança microbiológica é colocada em risco quando a equipe de enfermagem, durante o processo de administração de medicação, deixa de realizar medidas assépticas essenciais, como a higienização das mãos e a desinfecção de frascos e ampolas^(21)^. Nesse contexto, cabe ao enfermeiro o papel crucial de implementar estratégias contínuas de capacitação para sua equipe. É fundamental manter uma postura vigilante e proativa, mobilizando esforços coletivos para reduzir riscos e evitar falhas na assistência à saúde.

Observou-se também que nenhum profissional adotou conduta adequada nos cinco episódios observados nas medidas assépticas de desinfecção da ampola ou frasco-ampola e desinfecção do *hub* do cateter. Essa constatação é preocupante, considerando que a manipulação inadequada desses componentes pode favorecer a introdução de microrganismos na corrente sanguínea, contribuindo para o desenvolvimento de infecções primárias de corrente sanguínea e sepse. Evidências apontam que a falha na desinfecção adequada do *hub* do cateter está entre os principais fatores de risco para infecções associadas a cateteres venosos centrais, sendo considerada uma etapa crítica na prevenção desse tipo de infecção^(10)^.

A desinfecção inconforme dessas superfícies pode ser um ponto crítico no controle de infecções associadas ao cuidado endovenoso, como infecções relacionadas a cateteres. A literatura aponta que a desinfecção adequada de frascos e materiais utilizados em procedimentos invasivos é essencial para reduzir o risco de infecção, e a ausência de práticas adequadas sugere uma lacuna significativa nas práticas de controle de infecção nesse ambiente^(21)^.

Por sua vez, mais de 80% dos participantes praticaram seis medidas assépticas de maneira inconforme, o que levanta preocupações sobre a adesão dos profissionais às recomendações de controle de infecção. A implementação de treinamentos mais frequentes e a realização de auditorias podem ser necessárias para melhorar a adesão a esses protocolos.

É consenso na literatura que as superfícies clínicas em UTIs são potencialmente contaminadas por diversos patógenos, como *Enterococcus* resistente à vancomicina, *S. aureus* resistente à meticilina, *Clostridium difficile* e *Acinetobacter* sp., microrganismos capazes de resistir no ambiente mesmo após a limpeza terminal dos leitos^(3)^. Estudo prospectivo utilizando métodos microbiológicos evidenciou a presença de microrganismos em 81,1% das amostras coletadas após a limpeza terminal nos leitos de UTI, sendo 38,8% bacilos Gram-negativos^(2)^. Portanto, pode-se afirmar que as superfícies são potencialmente colonizadas por patógenos e inferir que o toque em superfícies clínicas por profissionais de saúde pode aumentar o risco de infecção cruzada durante a hospitalização^(22)^.

Neste estudo, foram registrados 1.083 episódios de toque das mãos dos profissionais em superfícies ambientais, com maior frequência em dispositivos críticos, como bomba de infusão (29,1%) e equipos (28,1%), que, juntos, representaram mais da metade dos contatos observados. Entretanto, mais importante do que o número absoluto de toques é que muitos desses contatos ocorreram sem a adoção das precauções assépticas recomendadas, o que pode aumentar significativamente o risco de contaminação cruzada durante a administração intravenosa de medicamentos.

O número médio de toques em superfícies clínicas por episódio de administração foi de oito vezes, valor relativamente elevado que aponta o ambiente de medicação como um ponto crítico para transmissão de patógenos. Esse cenário reforça a necessidade de políticas rigorosas de controle, com ênfase na higienização das superfícies manipuladas e na conscientização dos profissionais sobre as práticas adequadas.

A elevada frequência de toques em superfícies próximas ao paciente pode ser explicada por diversos fatores, como a sobrecarga de trabalho, que pode comprometer a adesão aos protocolos recomendados, dificultando a execução correta das ações de prevenção de infecções^(23)^. Ademais, o conhecimento insuficiente sobre práticas assépticas também contribui para essa realidade. Estudo realizado em UTI com equipes médica e de enfermagem evidenciou conhecimento limitado quanto às medidas consideradas padrão-ouro na prevenção de infecções da corrente sanguínea relacionadas ao cateter venoso central^(24)^.

As inconformidades nas medidas assépticas observadas são superiores às encontradas em estudo realizado no Marrocos^(25)^, que revelou o não cumprimento dos padrões de assepsia. Nesse estudo, 14,3% dos profissionais não higienizam as mãos antes e depois dos procedimentos; 7% não utilizavam luvas durante o atendimento; e 5,6% não trocavam as luvas entre os tratamentos. Essas práticas podem favorecer a ocorrência de infecções hospitalares, piorar o estado clínico dos pacientes, aumentar os custos de internação e elevar o risco de morte durante o período hospitalar^(2,15)^.

Apesar da elevada experiência profissional encontrada neste estudo, observou-se baixa adesão a práticas recomendadas para prevenção de infecções. Torna-se necessário investir em estratégias que sensibilizem a equipe quanto à importância da adesão às medidas assépticas e ao papel das superfícies ambientais na cadeia de transmissão de microrganismos.

### Limitações do estudo

Este estudo teve como limitações o tamanho da amostra e a condução em uma única UTI de um hospital universitário específico, o que restringe a generalização dos resultados para outras unidades ou contextos de cuidados de saúde. Apesar das ações de mitigação, o “Efeito Hawthorne” - o efeito da observação sobre o comportamento dos participantes, caracterizado como viés de reatividade - pode ter ocorrido. Assim, infere-se que a prática dos participantes possa ser de qualidade inferior à observada nos resultados. Para minimizar esse efeito, os profissionais foram observados durante cinco procedimentos, em cinco dias distintos, por um observador independente e não pertencente ao serviço.

Diante disso, pesquisas futuras devem explorar o impacto de intervenções multimodais, incluindo reestruturação logística, educacional e organizacional, assim como programas de melhoria de qualidade que abordem a problemática das superfícies clínicas como potenciais reservatórios de microrganismos. Além disso, é importante implementar indicadores de conformidade na administração de medicamentos via endovenosa. Essas pesquisas também devem levar em consideração as características e necessidades regionais, com amostras maiores, em locais diversos, e com desenhos quase-experimentais.

### Contribuições para as áreas da enfermagem e saúde

Os resultados deste estudo oferecem suporte para implementação de programas de melhoria da qualidade. Recomendam-se a expansão das políticas de educação permanente, revisão dos protocolos institucionais, treinamento contínuo das equipes, monitoramento e fornecimento de feedback sobre os resultados^(26)^. Destaca-se, ainda, o papel estratégico do enfermeiro líder de equipe na promoção da adesão às práticas baseadas em evidências e na consolidação de uma cultura de segurança no cuidado prestado pela equipe de enfermagem.

## CONCLUSÕES

Este estudo revelou que diversas barreiras de segurança essenciais para prevenção de IRAS apresentaram não conformidade durante a administração endovenosa de medicamentos por profissionais de enfermagem, o que pode comprometer a segurança do paciente e resultar em complicações graves. Sugerem-se a implementação de um programa de gerenciamento de cuidados e a realização de avaliações periódicas da prática de administração de medicamentos. Dessa forma, pode-se contribuir para ampliar a adesão às medidas assépticas e reduzir a frequência de toques desnecessários nas superfícies potencialmente contaminadas na administração de medicamentos e outros procedimentos de enfermagem.

## Data Availability

Os dados de pesquisa só estão disponíveis mediante solicitação.
